# An Unusual Presentation of 46,XY Pure Gonadal Dysgenesis: Spontaneous Breast Development and Menstruation

**DOI:** 10.4274/jcrpe.1919

**Published:** 2015-06-03

**Authors:** Gönül Çatlı, Caner Alparslan, P. Şule Can, Sinem Akbay, Sefa Kelekçi, Tahir Atik, Berk Özyılmaz, Bumin N. Dündar

**Affiliations:** 1 Tepecik Training and Research Hospital, Clinic of Pediatric Endocrinology, İzmir, Turkey; 2 Katip Çelebi University Faculty of Medicine, Department of Obstetrics and Gynecology, İzmir, Turkey; 3 Ege University Faculty of Medicine, Department of Medical Genetics, İzmir, Turkey; 4 Tepecik Training and Research Hospital, Clinic of Medical Genetics, İzmir, Turkey; 5 Katip Çelebi University Faculty of Medicine, Department of Pediatric Endocrinology, İzmir, Turkey

**Keywords:** 46, XY pure gonadal dysgenesis, breast development, amenorrhea, menstruation

## Abstract

46,XY pure gonadal dysgenesis (Swyer syndrome) is characterized by normal female genitalia at birth. It usually first becomes apparent in adolescence with delayed puberty and amenorrhea. Rarely, patients can present with spontaneous breast development and/or menstruation. A fifteen-year-old girl presented to our clinic with the complaint of primary amenorrhea. On physical examination, her external genitals were completely female. Breast development and pubic hair were compatible with Tanner stage V. Hormonal evaluation revealed a hypergonadotropic state despite a normal estrogen level. Chromosome analysis revealed a 46,XY karyotype. Pelvic ultrasonography showed small gonads and a normal sized uterus for age. SRY gene expression was confirmed by multiplex polymerase chain reaction. Direct sequencing on genomic DNA did not reveal a mutation in the SRY, SF1 and WT1 genes. After the diagnosis of Swyer syndrome was made, the patient started to have spontaneous menstrual cycles and therefore failed to attend her follow-up visits. After nine months, the patient underwent diagnostic laparoscopy. Frozen examination of multiple biopsies from gonad tissues revealed gonadoblastoma. With this report, we emphasize the importance of performing karyotype analysis, which is diagnostic for Swyer syndrome, in all cases with primary or secondary amenorrhea even in the presence of normal breast development. We also suggest that normal pubertal development in patients with Swyer syndrome may be associated with the presence of a hormonally active tumor.

## INTRODUCTION

46,XY pure gonadal dysgenesis (Swyer syndrome) is characterized by normal female genitalia at birth, underdeveloped Müllerian structures and fibrotic, primitive, non-productive gonads (1,2). Its incidence is estimated to be 1:80 000 births (3). Patients are usually diagnosed at adolescence with delayed puberty and primary amenorrhea (1,3,4). Classically, hypergonadotropic hypogonadism leads to absence of spontaneous breast development and of menstruation. However, we encountered reports of five cases with spontaneous breast development (4,5,6,7,8) and four cases with spontaneous menstrual cycles (4,7,9,10) in the literature.

In this report, we present the case of an apparently female adolescent with Swyer syndrome who had spontaneous breast development and menstruation considered to be due to the active hormone secretion from gonadoblastoma.

## CASE REPORT

**Figure 1:**
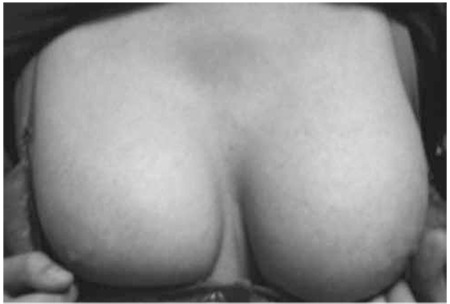
Bilateral Tanner stage V breast development.

**Figure 2:**
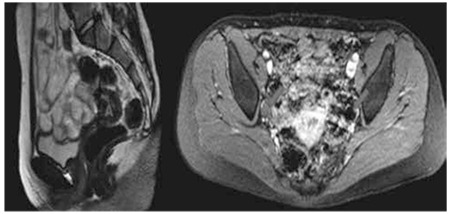
Magnetic resonance imaging of uterus (A) and gonads (red arrows).

A fifteen-year-old girl presented to our clinic with the complaint of primary amenorrhea. In her medical history, thelarche and pubarche were reported to occur at ages of 10 and 11 years, respectively. Family history was unremarkable and the parents were not related. On physical examination, weight was 55.2 kg [0.25 standard deviation (SD) for age and sex]; height was 165.5 cm (0.95 SD) and body mass index was 20.1 kg/m2 (0.10 SD). Her external genitalia were completely female. She had breast development and pubic hair of Tanner stage V (Figure 1). The rest of the physical examination was normal with no apparent somatic abnormalities and no clitoral enlargement or any other evidence of virilization. Laboratory findings revealed normal biochemistry. Hormone levels were as follows; follicle stimulating hormone, 121 mIU/mL (normal, 0.3-10); luteinizing hormone, 13 mIU/mL (normal, 0.3-31); estradiol, 66 pg/mL (normal, 15-350); total testosterone, <20 ng/dL (normal, 15-181); prolactin, 5.3 ng/mL (normal, 1.9-25); beta human chorionic gonadotropin, <1 mIU/mL (normal, 0-10); adrenocorticotropic hormone, 50.3 pg/mL (normal, 0-46) and cortisol, 14 µg/dL (normal, 5-25). Her bone age was consistent with 13.5 years according to the Greulich & Pyle method. Pelvic ultrasound revealed a right gonad of 1.4 cm3 and a left gonad of 2.4 cm3 in volume, with a uterus of 60x25x23 mm in size and an endometrial thickness of 7 mm. In magnetic resonance imaging, the gonads were observed to be smaller than normal. The uterus was of normal size and there was no evidence of a tumor (Figure 2). Chromosome analysis revealed a 46,XY karyotype (30 metaphase cells counted). SRY gene expression was confirmed by multiplex polymerase chain reaction. Direct sequencing on genomic DNA did not reveal a mutation in the SRY, SF1 and Wilms’ tumor 1 (WT1) genes. After the diagnosis of Swyer syndrome was made, the patient started to have spontaneous but irregular vaginal bleedings and therefore, she decided to quit her scheduled follow-up visits. After nine months, when she returned for a follow-up visit, the patient underwent a diagnostic laparoscopy. Frozen examination of multiple biopsies from gonad tissues revealed gonadoblastoma. Thus, the resection of the primary lesions and proper surgical staging (peritoneal washings, omental and peritoneal biopsies) were done to determine the presence of any occult metastasis. The uterus and other pelvic organs, except for the small streak gonads, were all normal and no palpable paraaortic or pelvic lymph nodes were detected. Histopathological examination of the dysgenetic gonads revealed bilateral pure gonadoblastoma and absence of follicular structures while the stroma was in a form resembling an ovarian structure. The patient underwent combined hormone replacement therapy with estrogen and progesterone, which provided regular monthly vaginal bleedings.

## DISCUSSION

Due to the absence of hormonal activity or reproductive potential of dysgenetic gonads, Swyer syndrome usually becomes apparent first in adolescence with delayed puberty and primary amenorrhea (1,11,12). The lack of adequate estrogen secretion almost always leads to a limitation in the development of secondary sexual characteristics. Similar to the many earlier reports of Swyer syndrome, our patient had primary amenorrhea with high gonadotropin levels and small ovaries without any Turner stigmata; however, normal estrogen levels, stage V breast development and spontaneous menstrual cycles that developed during follow-up were quite different and noteworthy. To our knowledge, spontaneous breast development was reported in only five cases with Swyer syndrome (4,5,6,7,8). Normal estrogen levels in patients with gonadal dysgenesis were hypothetically attributed to several mechanisms including i) production of estrogens by streak gonads at the time of puberty, ii) peripheral conversion of androgens to estrogens and iii) increased sensitivity of breast tissue to estrogens (4,8,13,14). Additionally, it has been reported that both male and female sex steroids may have been produced by gonadoblastoma (15) and that gonadoblastoma is often the source of hormones in females with the 46,XY karyotype (10,16). Joki-Erkkilä et al (17) reported sexual maturation in a 16-year-old adolescent girl with 46,XY karyotype and gonadoblastoma present in the dysgenetic gonads. Zieliñska et al (14) reported hormonal activity of gonadoblastoma in 3 patients with Swyer syndrome. Also, four cases with spontaneous menstruation due to neoplastic estrogen secretion were reported (4,7,9,10). We were not able to detect the exact mechanism for normal estrogen level, breast development, and spontaneous menstruation in our patient. However, it is possible that estrogen was secreted from the incipient gonadoblastoma and that this constant estrogen secretion led to irregular withdrawal bleeding rather than cyclic menstrual bleeding.

Neoplastic transformation of dysgenetic gonads can be seen in 15-35% of individuals with Swyer syndrome (18). Gonadoblastoma is the most frequent neoplasm associated with gonadal dysgenesis and is a benign tumor with no metastatic potential (1,3,19,20,21). However, it can transform to germ cell malignancies, such as dysgerminoma, teratoma, embryonal carcinoma or endodermal sinus tumor (3,9,13,18,20,21). Due to the high incidence of gonadoblastoma and germ cell malignancies, the current practice is to perform gonadectomy once the diagnosis of Swyer syndrome is made (22). In our case, surgical intervention led to the diagnosis of bilateral gonadoblastoma without any germ cell malignancies. We suggest that the presence of gonadoblastoma, which is hormonally active in secreting estrogens, may have masked gonadal dysgenesis and delayed the diagnosis.

Although our understanding of molecular determination of 46,XY pure gonadal dysgenesis has improved, in approximately 50% of the cases, the underlying genetic cause remains unknown (11). SRY (sex-determining region on the Y chromosome, MIM 480000, NM_005634) gene, which is located in the short arm of the Y chromosome (Yp11.3), is the best-defined gene in the differentiation of the bipotential gonad into a testis. However, mutations and deletions in SRY can be detected in only 10-20% of patients with Swyer syndrome (1,8). Mutations in the nuclear receptor subfamily 5 group A member 1/SF1 gene, WT1 gene, SOX9, desert hedgehog, mitogen-activated protein kinase 1, deletion of 9p24.3 and 10q26.1 and duplication of Xp21.2 are also related with 46,XY pure gonadal dysgenesis (23). In the current patient, chromosome analysis revealed a 46,XY karyotype. SRY gene expression was confirmed by multiplex polymerase chain reaction. Direct sequencing on genomic DNA did not reveal a mutation in the SRY, SF1 or WT1 genes. Molecular analysis of other genes could not be obtained.

Swyer syndrome is a rare condition, which can be easily diagnosed if the patients present with classical symptoms. However, patients can also present with spontaneous breast development and/or menstruation, which may delay the diagnosis. Because of the high incidence of neoplastic transformation and tendency of germ cell tumors to grow and metastasize rapidly, this delay may cause a poor prognosis and reduced survival rates. With this report, we emphasize the importance of performing karyotype analysis, which is diagnostic for Swyer syndrome, in all cases with primary or secondary amenorrhea even in the presence of normal breast development. We also suggest that normal pubertal development in patients with Swyer syndrome may be associated with the presence of a hormonally active tumor.
